# Host–Pathogen Crosstalk in Pediatric Peritoneal Dialysis-Associated Peritonitis: Molecular Mechanisms Driving Peritoneal Membrane Remodeling

**DOI:** 10.3390/ijms27073132

**Published:** 2026-03-30

**Authors:** John Dotis, Elias Iosifids, Charalampos Antachopoulos

**Affiliations:** Third Department of Pediatrics, Hippokration Hospital, Aristotle University of Thessaloniki, 54642 Thessaloniki, Greece; iosifidish@gmail.com (E.I.); antachop@auth.gr (C.A.)

**Keywords:** pediatric peritoneal dialysis, peritoneal dialysis-associated peritonitis, host–pathogen interactions, biofilm biology, mesothelial–mesenchymal transition, peritoneal fibrosis, inflammasome signaling

## Abstract

Peritoneal dialysis (PD)-associated peritonitis in children represents a complex interplay between microbial virulence, host immune activation and progressive peritoneal membrane remodeling. It should not be viewed solely as an acute infectious episode, but as a process unfolding within a chronically conditioned immune environment shaped by prolonged exposure to glucose-based dialysis solutions, oxidative stress and persistent biofilm formation on the Tenckhoff catheter. Mesothelial cells act as immunologically active sentinel cells, recognizing pathogen-associated molecular patterns through Toll-like receptors and related innate pathways. Subsequent activation of nuclear factor kappa B, inflammasome signaling and neutrophil extracellular trap formation further amplifies local inflammatory responses. Repeated inflammatory stimulation promotes mesothelial–mesenchymal transition, angiogenesis and extracellular matrix deposition driven by transforming growth factor beta 1 and interconnected profibrotic networks. In pediatric patients, prolonged PD vintage during critical stages of growth may intensify cumulative structural injury and increase the risk of ultrafiltration failure or encapsulating peritoneal sclerosis. Emerging strategies targeting inflammation, fibrosis and biofilm persistence, together with earlier molecular risk detection, may support preservation of the peritoneal membrane. A unified host–pathogen framework may therefore deepen pathophysiological insight and facilitate more individualized therapeutic strategies in pediatric PD.

## 1. Introduction

Peritoneal dialysis (PD) constitutes a principal modality of renal replacement therapy for children with end-stage kidney disease and remains particularly suited to long-term management in this population. Its home-based nature, together with the capacity to maintain residual renal function and provide adaptable treatment schedules, makes PD especially advantageous for pediatric care.

The durability of this therapy, however, depends largely on the long-term integrity of the peritoneal membrane [1,2]. Among the complications affecting membrane integrity, infectious episodes of peritonitis represent a major determinant of adverse outcomes [3]. Beyond the immediate clinical consequences of infection, recurrent or severe peritonitis initiates interconnected inflammatory and profibrotic processes driven by dynamic host–pathogen interactions within a chronically conditioned peritoneal environment. Elucidating these host–microbe interactions is therefore critical for understanding the mechanisms that drive peritoneal membrane injury and remodeling.

In this review, we synthesize current insights into pathogen-induced immune responses, key inflammatory signaling pathways and the cellular processes underlying structural remodeling of the peritoneum, with particular emphasis on their relevance to pediatric PD.

### 1.1. The Peritoneal Cavity as a Bioactive Immunological Compartment

The long-term effectiveness of PD is intrinsically linked to the preservation of peritoneal membrane structure and function. Although traditionally viewed as a semi-permeable surface for solute and fluid exchange, the peritoneum increasingly emerges as a biologically active tissue with complex regulatory properties. The mesothelial monolayer does not merely serve as a physical boundary but constitutes an active participant in host defense, sensing microbial and chemical stressors and coordinating downstream inflammatory responses during peritonitis [1].

Sustained exposure to dialysis solutions introduces repetitive biochemical stress that progressively alters cellular homeostasis. Rather than representing isolated injury events, these stimuli initiate interconnected molecular responses involving inflammatory mediators, oxidative stress pathways and fibrotic signaling networks [4]. Within this context, the peritoneum functions as a biologically active immunological interface whose structural integrity is continuously shaped by environmental and inflammatory cues.

The peritoneal cavity also contains resident immune populations and stromal cells that operate within a tightly regulated microenvironment. Continuous dialysate exposure may provoke sterile inflammatory activation characterized by reciprocal communication between immune and structural compartments [5]. Over time, this persistent low-grade activation contributes to vasculopathic alterations and progressive fibrotic remodeling, processes in which oxidative stress appears to play a contributory role [2]. Collectively, these observations support a conceptual shift toward viewing the peritoneum as an adaptive immunological interface rather than a passive dialysis membrane.

### 1.2. Paradigm Shift

Peritonitis remains a principal complication of PD and a major determinant of technique discontinuation. Historically framed as an acute infectious condition requiring prompt antimicrobial therapy, peritonitis is now recognized as an event with broader biological implications. Each episode unfolds within a peritoneal environment already conditioned by chronic dialysate exposure, thereby amplifying preexisting inflammatory pathways and accelerating structural remodeling processes [4].

In parallel, dialysis solutions themselves contribute to sterile inflammatory signaling that promotes immune cell recruitment and sustains cytokine production [5]. Recurrent inflammatory stimulation activates transcriptional programs linked to extracellular matrix synthesis and fibrogenesis, ultimately altering membrane architecture [6]. These processes are further elaborated on in later mechanistic sections. High-glucose formulations further intensify these responses by reinforcing transforming growth factor beta (TGF-β) dependent pathways and other profibrotic mediators [7]. A failure of timely inflammatory resolution may represent an early mechanistic gateway linking acute peritoneal inflammation to subsequent fibrotic remodeling. Consequently, membrane injury should be understood not solely as the direct effect of microbial invasion but as the cumulative result of dysregulated host responses occurring within a chronically primed immunological environment.

### 1.3. Pediatric Vulnerability

Children undergoing long-term PD constitute a distinct clinical population in whom developmental factors intersect with chronic inflammatory exposure. Although advances in infection prevention have reduced peritonitis incidence, the combined burden of prolonged dialysate exposure and repeated inflammatory activation continues to shape long term membrane outcomes [8]. Progressive structural and functional alterations of the peritoneal membrane remain central challenges in pediatric technique survival.

Emerging evidence indicates that inflammatory and profibrotic mediators may persist locally even after apparent clinical resolution of infection [1]. Recurrent stimulation of cytokine networks and matrix producing pathways fosters gradual architectural transformation of the peritoneal membrane [6]. Chronic exposure to high glucose solutions further sustains TGF-β signaling and related fibrotic mechanisms [7].

In the pediatric setting, where dialysis often coincides with critical phases of growth and developmental maturation, the cumulative biological impact of sustained inflammatory signaling may be particularly consequential. However, much of the mechanistic evidence currently available has been derived primarily from adult populations or from heterogeneous cohorts that combine adult and pediatric patients, which may limit the direct extrapolation of these findings to children. Rare but severe outcomes such as encapsulating peritoneal sclerosis (EPS) underscore the potential long-term consequences of unresolved structural injury [8].

### 1.4. Gap in Literature

Recent scholarship has elucidated multiple components of immune-mediated peritoneal injury, including sterile inflammatory signaling, stromal immune interactions, oxidative stress responses and profibrotic cascades [2,4]. Mechanistic investigations have further highlighted the interplay between TGF-β1 and vascular endothelial growth factor A (VEGF-A) in promoting fibrosis and neoangiogenesis associated with ultrafiltration dysfunction [9].

Despite these advances, current literature often addresses these processes in isolation. Although several mechanistic frameworks describing peritoneal inflammation and fibrosis have been proposed, a fully integrated model that connects innate immune activation, pathogen-driven inflammatory amplification and chronic biochemical conditioning by dialysis fluids to progressive structural remodeling remains incompletely developed [4]. Advanced pathological manifestations such as EPS exemplify the cumulative trajectory of unresolved injury [10]. The development of a unified conceptual framework linking host response dynamics to long-term membrane transformation may therefore refine our understanding of the mechanisms driving membrane injury and support future translational strategies.

## 2. The Peritoneal Membrane as an Active Immunological Interface

### 2.1. Mesothelial Cells as Innate Immune Sensors

The mesothelial monolayer constitutes the first immunologically active barrier encountering microbial molecular patterns derived from bacteria and fungi within the peritoneal cavity. It has been demonstrated that human peritoneal mesothelial cells express multiple Toll-like receptors (TLRs), including TLR1 through TLR6, with TLR2 playing a particularly prominent functional role [11]. Mesothelial TLR2, and to a lesser extent TLR4, contributes to the initiation and amplification of proinflammatory and profibrotic signaling cascades following bacterial exposure in the peritoneal environment [12]. It may be hypothesized that developmental differences in innate immune signaling could modulate TLR responsiveness in pediatric patients, potentially influencing the magnitude and duration of downstream inflammatory activation.

Upon engagement of TLR2 by Gram-positive bacterial components or TLR4 by lipopolysaccharide (LPS) derived from Gram-negative bacteria, intracellular signaling cascades are triggered, leading to activation of nuclear factor kappa B (NF-κB) and multiple mitogen-activated protein kinases (MAPKs). In human peritoneal mesothelial cells, ligand stimulation induces phosphorylation of extracellular signal–regulated kinases 1 and 2 (ERK1/2), c-Jun N-terminal kinase (JNK), p38 MAPK and NF-κB p65, which collectively drive the transcriptional upregulation of interleukin-1β (IL-1β) and tumor necrosis factor-α (TNF-α) [13]. These pathways illustrate how receptor engagement translates into a coordinated inflammatory gene expression profile. Additionally, TLR2-dependent signaling has been implicated in infection-associated fibrotic remodeling [12].

In the context of fungal pathogens, β-glucan motifs are detected primarily through the C-type lectin receptor Dectin-1 (CLEC7A). The structural organization of fungal β-glucans promotes receptor clustering at the membrane level, facilitating signal amplification through activation-dependent oligomerization mechanisms [14]. Collectively, these mechanisms position mesothelial cells as active immunological sentinels integrating microbial signals into coordinated inflammatory and remodeling responses. Whether these sensing mechanisms exhibit age-dependent modulation in children remains to be clarified and represents an area for future investigation.

### 2.2. Professional Phagocytes

In addition to mesothelial cells, recruited neutrophils and resident peritoneal macrophages represent the principal effector populations responsible for early containment of infection. During peritoneal inflammatory injury, TLR2-mediated signaling in macrophages substantially enhances the production of cytokines and mediators that simultaneously sustain inflammation and promote tissue remodeling, thereby linking innate immune sensing with structural alterations [12]. TNF-α further amplifies this inflammatory circuit and supports continued leukocyte recruitment.

Neutrophils are rapidly mobilized into the peritoneal cavity in response to infectious and sterile inflammatory stimuli. Among their antimicrobial strategies is the formation of neutrophil extracellular traps (NETs), a process characterized by extracellular DNA release and deposition of granular enzymes. In pediatric patients undergoing chronic PD, NET structures have been identified in dialysate fluid and peritoneal biopsies, where their presence correlates with increased submesothelial thickening and enhanced microvascular density [15]. These observations support a mechanistic association between persistent NET formation and structural membrane remodeling. It is plausible that age-related differences in neutrophil function or NET regulation may influence the balance between antimicrobial defense and tissue injury in pediatric patients.

Clinical observations reinforce this association, as elevated levels of peritoneal NET markers correlate with accelerated solute transport rates and a higher probability of technique failure [16]. It is essential to distinguish NETosis, a nicotinamide adenine dinucleotide phosphate (NADPH) oxidase-dependent regulated cell death pathway, from related processes such as defective mitophagy or leukotoxic hypercitrullination, which may also result in extracellular DNA release but arise through distinct biological mechanisms [17]. This distinction is relevant when interpreting inflammatory biomarkers in peritoneal dialysate. Overall, professional phagocytes exert a dual role, being essential for antimicrobial defense while potentially contributing to ongoing structural injury when persistently activated.

### 2.3. Chronic Priming by PD Fluids

Long-term exposure to PD solutions imposes repetitive biochemical stress on the peritoneal membrane, leading to adaptive yet potentially maladaptive alterations in innate immune responsiveness. Experimental data indicate that glucose-based dialysates can downregulate TLR2 and TLR4 expression in mesothelial cells, thereby attenuating subsequent activation of MAPK and NF-κB signaling pathways upon pathogen challenge [13]. This modulation promotes a state of relative immunological hyporesponsiveness to pathogen-associated molecular patterns.

At the same time, sterile inflammatory signaling driven by endogenous danger-associated molecular patterns and metabolic stress contributes to progressive fibrotic transformation during long-term dialysis [18]. Continuous exposure to uremic toxins and bioincompatible solutions appears to maintain baseline TLR activation even in the absence of overt infection, reinforcing the interplay between innate immune signaling and membrane remodeling. Consequently, chronic dialysis establishes a persistent low-grade inflammatory milieu within mesothelial and immune cell populations.

**Figure 1:**
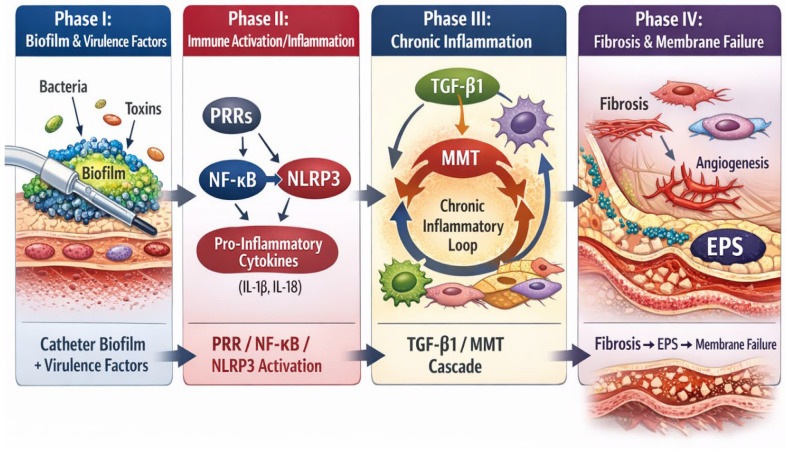
This figure illustrates the sequential biological progression from catheter-associated biofilm formation and pathogen virulence (Phase I) to pattern recognition receptor-mediated activation and inflammasome signaling (Phase II), followed by chronic inflammatory amplification and transforming growth factor beta 1-driven mesothelial–mesenchymal transition (Phase III). The final phase (Phase IV) reflects progressive fibrosis, angiogenesis, and structural reorganization culminating in ultrafiltration failure, fast transport phenotype, and encapsulating peritoneal sclerosis. The model integrates microbial persistence with host immune dysregulation as converging drivers of membrane failure.

Under these primed conditions, subsequent infectious stimuli occur against an already activated background, effectively lowering the threshold for fibrotic progression and structural deterioration [4,7]. In pediatric patients, prolonged exposure during critical developmental stages may further modulate this priming effect, potentially amplifying susceptibility to long-term structural remodeling. The sequential transition from innate immune activation to progressive structural remodeling is conceptually summarized in Figure 1.

## 3. Microbial Virulence and Pathogen-Specific Molecular Signatures

### 3.1. Gram-Positive Bacteria

PD-associated peritonitis does not represent a uniform inflammatory event. The biological behavior of individual pathogens determines how the chronically primed peritoneal membrane responds. Gram-positive organisms remain the most frequent pathogens in pediatric PD-associated peritonitis [3]. These bacteria predominantly activate TLR 2 through recognition of lipoteichoic acid and bacterial lipoproteins [19]. In human peritoneal mesothelial cells, TLR2 engagement promotes NF-κB-dependent cytokine production and neutrophil recruitment, contributing to early immune containment [11].

Although inflammatory activation can be substantial, it frequently remains compartmentalized and responsive to antimicrobial therapy. A key virulence feature of many Gram-positive organisms is their ability to form biofilms on the Tenckhoff catheter, enabling bacterial persistence within an extracellular matrix that reduces antibiotic penetration and sustains intermittent antigenic stimulation [20]. Consistent with this biological profile, the currently available data appear to indicate a relatively higher membrane salvage potential and lower mortality compared with fungal and mycobacterial infections [21,22]. In this context, structural injury is typically linked to recurrence and chronic biofilm-mediated stimulation rather than intrinsic tissue invasiveness.

### 3.2. Gram-Negative Bacteria

In contrast, Gram-negative pathogens elicit a distinct inflammatory signature driven by lipopolysaccharide-mediated activation of Toll-like receptor 4 (TLR4) [23]. Engagement of TLR4 initiates downstream signaling through the adaptor molecules myeloid differentiation primary response 88 (MyD88) and Toll/Interleukin-1 receptor (TIR) domain-containing adaptor inducing interferon beta (TRIF), leading to enhanced cytokine production, endothelial activation, and increased vascular permeability within the peritoneal membrane. In a membrane already conditioned by chronic dialysate exposure and baseline inflammatory priming, endotoxin-driven signaling may therefore reach a higher inflammatory amplitude than that observed in typical Gram-positive infections [23].

Clinical data position Gram-negative pathogens within an intermediate severity tier, with generally favorable survival but variable catheter removal rates [3]. Membrane injury in this setting reflects intensified inflammatory amplification superimposed on a primed environment, with greater potential for acute functional disturbance yet often reversible structural impact when promptly controlled.

### 3.3. Mycobacteria

Different pathogen groups activate distinct innate immune pathways, generating pathogen-specific inflammatory signatures that influence the trajectory of peritoneal membrane injury. Mycobacterial infections represent a fundamentally different pathogenic paradigm characterized by intracellular persistence. Tuberculous and nontuberculous *Mycobacteria* survive within macrophages by interfering with phagosome maturation and resisting lysosomal degradation [24]. The host response is dominated by sustained T helper 1 (Th1)-driven signaling, with interferon gamma (IFN-γ) and TNF-α as central mediators [25].

Within the chronically exposed peritoneal environment of long-term dialysis, persistent cytokine production and prolonged antigenic stimulation tend to favor progressive remodeling rather than extensive acute necrosis. Pediatric cases of nontuberculous mycobacterial peritonitis, although uncommon, are frequently associated with prolonged therapy and high rates of catheter removal [22]. In addition, mycobacterial infections are consistently linked to technique failure despite relatively low infection-related mortality. Although pathophysiologically distinct from typical bacterial peritonitis, mycobacterial infections are difficult to eradicate, often requiring prolonged multidrug therapy and frequently culminating in catheter removal. The biological trajectory is therefore defined by chronic immune activation and gradual structural transformation of the membrane.

### 3.4. Fungal Pathogens

Fungal peritonitis in PD is predominantly attributed to *Candida* spp. among yeasts and to *Aspergillus* spp. among filamentous molds, with *Candida* spp. representing most cases across both pediatric and adult populations [3,21]. In contrast to bacterial pathogens, fungal peritonitis demonstrates distinct biological and clinical characteristics that may partly explain its less favorable outcomes.

Fungal cell wall components, particularly beta glucans, are recognized by pattern recognition receptors such as CLEC7A and TLRs, triggering coordinated innate immune signaling cascades that amplify inflammatory responses within the peritoneal cavity [26]. *Candida* spp. are polymorphic yeasts capable of transitioning between yeast and hyphal forms, a property that enhances adherence and interaction with mesothelial surfaces. Within the PD environment, biofilm formation on a Tenckhoff catheter represents a central pathogenic mechanism. Biofilm-associated pathogens exhibit enhanced antifungal tolerance and impaired immune clearance, frequently necessitating catheter removal despite systemic or intraperitoneal therapy [27]. This combination of immune activation and biofilm-associated persistence likely contributes to higher rates of technique failure compared with most bacterial infections. Consistent with this biological and clinical profile, current International Society for Peritoneal Dialysis guidelines recommend early catheter removal in cases of fungal peritonitis to improve outcomes [3].

In contrast, *Aspergillus* spp. exhibit a distinctly invasive growth pattern characterized by hyphal extension and structural tissue penetration. While early beta glucan recognition contributes to immune activation, established hyphal growth enables direct mesothelial invasion and angioinvasion, potentially leading to vascular thrombosis, ischemia and tissue necrosis [28,29]. Available systematic data suggest that *Aspergillus* peritonitis is associated with particularly high rates of catheter removal and infection-related mortality [21].

Overall, published case series and contemporary guideline-informed experience suggest that fungal infections occupy the higher-severity spectrum of PD-associated infections, largely reflecting high rates of catheter loss and adverse clinical outcomes [21]. The comparative immunobiological profiles of the different pathogen groups are summarized in Table 1.

Summary of the principal pattern-recognition receptors, downstream inflammatory pathways and structural consequences associated with major pathogen groups causing PD-associated peritonitis. Pathogen-specific immune activation profiles contribute to distinct patterns of inflammatory amplification, membrane remodeling and clinical outcomes [3,11,12,21,23,26].

Abbreviations: Toll-like receptor (TLR); nuclear factor kappa B (NF-κB); mitogen-activated protein kinases (MAPK); myeloid differentiation primary response 88 (MyD88); Toll/interleukin-1 receptor (TIR) domain-containing adaptor inducing interferon beta (TRIF); NOD-like receptor family; T helper 1 (Th1); interferon gamma (IFN-γ); tumor necrosis factor alpha (TNF-α); spleen tyrosine kinase (Syk); caspase recruitment domain family member 9 (CARD9).

## 4. Biofilm Biology and the Catheter Microenvironment

### 4.1. The Biofilm Matrix as a Structured Microbial Niche

Biofilm formation on the Tenckhoff catheter represents a critical interface between acute infectious events and longer-term membrane injury. Rather than existing as free-floating planktonic organisms, bacteria embedded within biofilms organize into structured microbial communities encased in a self-produced extracellular polymeric matrix. This organization permits stable colonization of localized niches while increasing tolerance to host immune defenses and antimicrobial [30]. In pediatric patients, differences in immune maturation and microbial exposure may influence early biofilm establishment and stability, although direct evidence remains limited.

The extracellular polymeric substance matrix is not simply a passive barrier. It is a dynamic, shared microenvironment composed of polysaccharides, proteins, lipids, amyloids, membrane vesicles and extracellular DNA that collectively maintain structural integrity and facilitate coordinated microbial behavior [31]. The matrix regulates diffusion of nutrients and signaling molecules, retains exoenzymes and creates microgradients that shape metabolic heterogeneity within the biofilm community.

Extracellular DNA, derived from bacterial lysis or regulated release, serves as an essential structural scaffold within many biofilms. Beyond its architectural role, it contributes to mechanical stability and intercellular cohesion [32]. Experimental degradation of extracellular DNA has been shown to destabilize biofilm architecture, underscoring its functional relevance rather than incidental presence.

Importantly, biofilms demonstrate coordinated spatial architecture and temporal adaptation that resemble multicellular developmental programs. The presence of localized metabolic specialization and regulated community behavior supports the view that biofilms function as structured biological systems, not merely as bacterial accumulations [33]. This structured organization enhances resilience and persistence, particularly in device-associated environments such as PD catheters.

### 4.2. The Catheter as a Foreign Body Interface

The Tenckhoff catheter introduces a permanent biomaterial surface into the peritoneal cavity, thereby altering local immune homeostasis. Biomaterials are known to provoke a spectrum of host responses, including inflammatory activation, foreign body reactions and varying degrees of fibrotic encapsulation, depending on their physicochemical properties and surface characteristics [34]. Even in the absence of overt infection, implanted materials may modify leukocyte behavior and cytokine profiles in their immediate vicinity. In children, developmental differences in immune reactivity and tissue repair may further modulate these host–device interactions.

Surface irregularities, protein adsorption layers and microtopography can facilitate early microbial adhesion. Once colonization is established, implant-associated immune modulation may impair efficient bacterial clearance, creating conditions favorable to biofilm maturation [35]. In this setting, pathogen adhesion factors and altered host responses operate in concert, enabling persistent colonization.

In the PD setting, the catheter functions not only as a device for dialysate exchange but also as a biologically active surface. It provides a microenvironment in which microbial biofilms coexist in direct proximity to macrophages, neutrophils and mesothelial cells. Continuous paracrine signaling between these compartments sustains local inflammatory activation even when systemic signs of infection have resolved [31,36].

### 4.3. Biofilm Persistence and Chronic Peritoneal Inflammation

In PD-associated infections, biofilms are increasingly recognized not only on catheter surfaces but also within adjacent tissues, where they may act as reservoirs of recurrent microbial stimulation and contribute to relapsing or catheter-related peritonitis [36]. The interaction between host cells, dialysis fluids and structured microbial communities generates a microenvironment that favors persistent, low-grade inflammatory activation rather than complete immune resolution. Whether this persistence is accentuated in pediatric patients due to prolonged device exposure during developmental stages remains an open question.

Repeated exposure to biofilm-derived pathogen-associated molecular patterns maintains TLR-mediated signaling in mesothelial cells and peritoneal macrophages. Over time, this sustained stimulation may shift the biological response from transient containment toward progressive tissue adaptation and remodeling. Experimental data indicate that activation of TLR 2 and TLR 4 contributes directly to profibrotic signaling pathways and peritoneal membrane transformation in the context of infection [12].

Importantly, modulation of TLR-mediated pathways has been associated with attenuation of infection-driven fibrotic responses without compromising antimicrobial defense mechanisms [12]. These observations reinforce the concept that biofilm persistence does not merely sustain infection but may actively participate in the gradual structural alteration of the peritoneal membrane [18]. In pediatric patients, developmental differences in immune regulation may further modulate the balance between microbial clearance and chronic inflammatory activation. Within this framework, persistent biofilm presence may act as a mechanistic link between recurrent peritonitis and progressive membrane remodeling, ultimately contributing to technique failure.

## 5. Inflammation and Peritoneal Remodeling: The Mesothelial–Mesenchymal Transition (MMT) Axis

### 5.1. TGF-β1 as a Central Regulatory Node

The shift from short-lived inflammatory signaling toward sustained structural reorganization represents a pivotal turning phase in the progressive decline of peritoneal membrane integrity. TGF-β1 functions as a central molecular orchestrator of this transition. Persistent inflammatory stimuli, including infectious episodes, biofilm retention, or chronic exposure to bioincompatible dialysate, enhance local TGF-β1 synthesis by both mesothelial cells and activated macrophages [7,9]. It may be hypothesized that developmental differences in cellular signaling and repair capacity could modulate TGF-β1-driven responses in pediatric patients, potentially influencing the trajectory of fibrotic remodeling.

Upon activation, TGF-β1 initiates MMT, a phenotypic reprogramming process marked by downregulation of epithelial adhesion molecules such as E-cadherin, upregulation of alpha smooth muscle actin, enhanced cellular motility and augmented extracellular matrix synthesis. These alterations are propagated through both Sma and Mad homology (Smad)-dependent transcriptional signaling and alternative Smad-independent cascades, including activation of MAPK pathways [6,7]. Whether the balance between these signaling pathways differs in the developing peritoneum remains unclear and warrants further investigation.

Emerging evidence suggests that mitochondrial impairment acts as a potentiating factor in this phenotypic conversion. Effluent-derived mesothelial cells obtained from patients undergoing PD exhibit elevated mitochondrial reactive oxygen species (ROS) generation and reduced mitochondrial membrane potential, particularly among cells displaying a fibroblast-like morphology [37]. Pharmacologic neutralization of mitochondrial-derived ROS has been shown to blunt TGF-β1-driven fibronectin upregulation, supporting a role for mitochondrial stress as a permissive amplifier of MMT. Age-related differences in mitochondrial resilience may therefore represent an additional layer of regulation in pediatric peritoneal remodeling.

Concurrently, endogenous antifibrotic control mechanisms may become progressively attenuated during long-term exposure to high-glucose dialysate solutions. C-Jun N-terminal kinase-associated leucine zipper protein (JLP) has been identified as a suppressive regulator of TGF-β signaling activity. Loss or downregulation of this factor intensifies high-glucose associated peritoneal fibrotic remodeling in experimental settings, further augmenting ΜΜΤ and Smad pathway activation [38]. These findings suggest that progressive remodeling reflects both sustained profibrotic signaling and impaired endogenous regulatory mechanisms.

### 5.2. Angiogenesis, Matrix Stiffness, and Stromal Feedback

Progressive fibrotic restructuring occurs in parallel with expanding neovascularization and measurable shifts in the biomechanical characteristics of the submesothelial matrix. TGF-β1 stimulates VEGF-A production, thereby integrating fibrotic remodeling and angiogenic expansion within an interconnected signaling framework [9]. Increased microvascular density modifies solute transport kinetics and contributes to the development of ultrafiltration failure. It is plausible that developmental differences in angiogenic regulation may influence the extent and functional impact of neovascularization in pediatric patients.

Beyond molecular signaling events, alterations in extracellular matrix composition reshape the physical properties of the peritoneal stroma. Accumulation of cross-linked collagen fibers and disruption of normal matrix architecture modify cellular mechanotransduction cues and influence macrophage polarization dynamics. Emerging evidence suggests that macrophage–stroma interactions are bidirectional, with increased matrix stiffness reinforcing pro-inflammatory and profibrotic macrophage phenotypes [39]. This interaction establishes a self-reinforcing circuit linking inflammation, matrix remodeling, and immune activation. Whether these feedback mechanisms are amplified or attenuated in the developing peritoneal environment remains to be clarified.

### 5.3. Progressive Structural Failure

With prolonged exposure to inflammatory and profibrotic stimuli, cumulative microarchitectural distortion becomes progressively apparent. Histologically, chronic and unresolved signaling activity is characterized by progressive submesothelial thickening, increased collagen deposition, vasculopathic remodeling and focal dystrophic calcification in advanced stages. EPS represents the terminal expression of this pathological continuum, characterized by extensive fibrotic encapsulation and pronounced vascular remodeling [10].

Collectively, available data support an integrated pathobiological model in which infection-associated inflammation, mitochondrial stress responses, failure of endogenous antifibrotic safeguards and dynamic macrophage–stroma crosstalk converge to drive sustained membrane remodeling. Within this framework, fibrosis reflects the cumulative outcome of persistent immune activation within a metabolically and mechanically altered microenvironment, rather than an isolated late-stage complication [6,37]. In pediatric patients, prolonged exposure during critical developmental periods may further modulate the threshold for irreversible structural damage. The integrated molecular axes connecting persistent inflammatory activation to progressive structural and functional membrane decline are comparatively outlined in Table 2.

Overview of key molecular axes connecting innate immune activation with progressive peritoneal membrane remodeling and functional decline in PD. These pathways illustrate how persistent inflammatory signaling may drive fibrosis, angiogenesis and dialysis technique dysfunction [6,7,9,11,12,15,36].

Abbreviations: Pattern-recognition receptors (PRR); nuclear factor kappa B (NF-κB); NOD-like receptor family pyrin domain-containing 3 (NLRP3); interleukin-1 beta (IL-1β); neutrophil extracellular traps (NET); transforming growth factor beta 1 (TGF-β1); mesothelial–mesenchymal transition (MMT); vascular endothelial growth factor (VEGF); hypoxia-inducible factor (HIF).

## 6. Pediatric-Specific Considerations

### 6.1. Developmental Immunity and Neutrophil Extracellular Trap (NET)-Driven Remodeling

Children receiving long-term PD represent a biologically distinct population in which immune system maturation coincides with continuous peritoneal exposure to dialysis-related stressors. According to recent international clinical practice guideline updates, infection-related complications remain a predominant contributor to morbidity and technique failure in pediatric PD [40]. Beyond the immediate consequences of acute infectious peritonitis, emerging evidence indicates that the pediatric peritoneum may undergo remodeling processes driven not only by clinically evident infection but also by sustained, low-grade sterile inflammatory pathways.

Recent biopsy-based evidence from the International Pediatric Peritoneal Biobank has shown that prolonged PD exposure in children correlates with increased microvessel density, submesothelial thickening and collagen deposition, accompanied by substantial accumulation of NET-associated markers within peritoneal tissue [15]. Elevated dialysate concentrations of cell-free DNA, citrullinated histone H3, neutrophil elastase and myeloperoxidase were found to correlate with histological structural alterations, while reduced DNase activity suggested impaired degradation and clearance of extracellular chromatin [15]. These findings reinforce the hypothesis that NETs extend beyond antimicrobial defense and actively contribute to the propagation of persistent inflammatory and profibrotic signaling within the pediatric peritoneal compartment.

In this context, developmental variability in innate immune responses may modulate both the intensity and duration of inflammatory activation. Even after apparent clinical resolution of peritonitis, inflammatory mediators may persist locally within the peritoneal cavity, thereby potentially reducing the threshold required to trigger subsequent remodeling cascades [1]. The pediatric membrane may therefore experience a process of cumulative immune conditioning, whereby repeated activation of neutrophil and macrophage pathways progressively accelerates structural adaptation and fibrotic transformation over time [1,6]. This concept supports the view that early-life inflammatory exposures may contribute to long-term structural vulnerability of the peritoneal membrane.

### 6.2. Dialysis Vintage and Cumulative Dialysate Exposure

Children frequently initiate PD early in life and may remain on therapy for extended periods prior to transplantation. Prolonged dialysis vintage has repeatedly been identified as a risk factor for advanced membrane complications, including EPS [41]. Although the overall incidence of pediatric EPS remains low, its association with long-term exposure highlights the clinical importance of cumulative peritoneal stress and injury.

The introduction of more biocompatible, neutral PD solutions with reduced glucose degradation products has been associated with a measurable reduction in both the incidence and severity of EPS across large national cohorts [42,43]. Collectively, these data strengthen the link between chronic biochemical exposure and late-stage membrane transformation. Current pathophysiological models conceptualize EPS not merely as excessive fibrosis but as the formation of a neo-membranous structure following sustained peritoneal injury [42]. This framework aligns with the broader model of progressive remodeling mediated by recurrent inflammatory and profibrotic signaling networks.

In pediatric populations, where dialysis exposure spans critical windows of somatic growth and immunological development, the interplay between dialysis vintage, recurrent inflammation and host susceptibility assumes pathophysiological significance. Despite advances in infection prevention, non-infectious membrane failure continues to represent a substantial and unresolved clinical challenge [8]. These observations underscore the importance of viewing dialysis vintage not merely as a temporal variable but as a cumulative biological modifier of membrane integrity.

### 6.3. Risk Stratification and Early Biological Signals

A central pediatric challenge lies in distinguishing reversible adaptive alterations from trajectories that progress toward irreversible structural membrane failure. Although rare, EPS represents the extreme endpoint of persistent inflammatory activation and maladaptive fibrotic remodeling [10,42]. Early identification of high-risk patients remains a critical unmet clinical objective.

The integration of tissue-based findings, dialysate biomarker profiling and registry-derived clinical data suggests that membrane vulnerability may reflect both cumulative environmental exposure and intrinsic host determinants. Recent guideline updates underscore the importance of structured longitudinal monitoring and continued investigation into predictive markers of peritoneal injury in children [40]. The growing recognition of neutrophil extracellular trap burden as a correlate of structural remodeling adds further support to the concept that molecular signatures detected in peritoneal effluent may serve as early indicators of subclinical injury and progressive membrane dysfunction [15].

**Figure 2:**
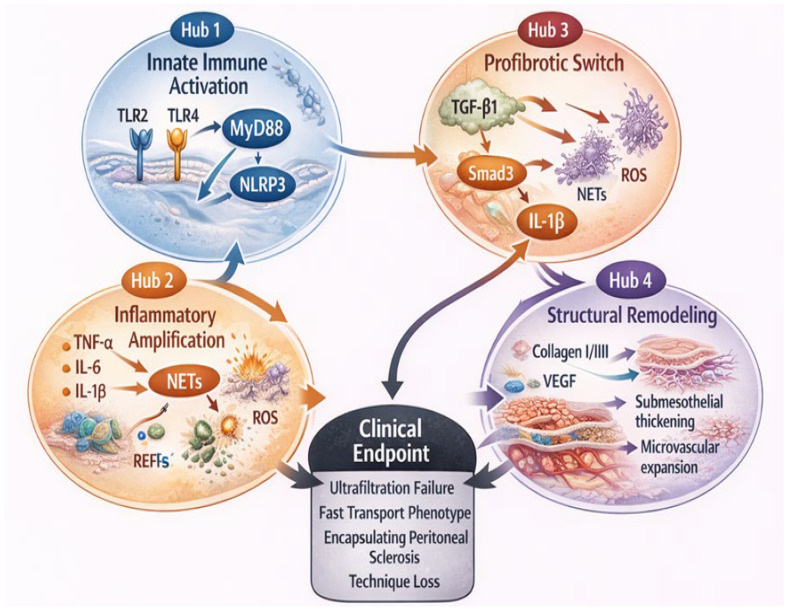
This figure illustrates the integrated molecular network linking innate immune activation to progressive peritoneal fibrosis in pediatric peritoneal dialysis. Hub 1 represents innate immune activation through Toll-like receptor 2 (TLR2) and Toll-like receptor 4 (TLR4) signaling, MyD88-dependent pathways, nuclear factor kappa B (NF-κB) activation, and NLRP3 inflammasome-mediated caspase-1 activation. Hub 2 illustrates inflammatory amplification via tumor necrosis factor alpha (TNF-α), interleukin-6 (IL-6), neutrophil extracellular traps (NETs), and reactive oxygen species (ROS). Hub 3 represents the profibrotic switch driven by transforming growth factor beta 1 (TGF-β1), Smad3 signaling, mesothelial–mesenchymal transition (MMT), extracellular vesicle-mediated signaling, and macrophage polarization. Hub 4 represents structural remodeling characterized by collagen type I/III deposition, vascular endothelial growth factor (VEGF)-mediated angiogenesis, submesothelial thickening, and microvascular expansion, ultimately leading to technique failure.

Taken together, pediatric PD should be conceptualized within a longitudinal developmental framework. Membrane remodeling in children reflects the convergence of immune maturation, cumulative dialysis exposure, biofilm-associated dynamics and recurrent inflammatory activation within a chronically conditioned peritoneal milieu. A deeper understanding of these pediatric-specific biological processes may facilitate earlier identification of patients at risk, enable targeted intervention and support long-term technique preservation. Future studies integrating longitudinal biomarker profiling with histological validation may clarify whether NET-related signatures can inform individualized membrane-preserving strategies in pediatric PD. The integrated molecular crosstalk network linking innate immune activation, inflammatory amplification, profibrotic transition and structural membrane remodeling is illustrated in Figure 2.

## 7. Translational Targets and Future Perspectives

### 7.1. Targeting Fibrogenic Signaling Networks

The transition from reversible inflammatory activation to sustained structural remodeling represents a decisive phase in peritoneal membrane failure. Rather than being driven by a single cytokine such as TGF-β1, fibrogenesis appears to arise from the convergence of inflammatory, metabolic and immune-mediated signaling pathways [6,7]. This conceptual framework highlights multiple potential points of therapeutic intervention.

Inflammasome activation functions as a central regulatory node. Inhibition of the NOD-like receptor family pyrin domain-containing 3 (NLRP3) inflammasome and downstream caspase-1-dependent IL-1β maturation attenuated collagen deposition, angiogenesis, and epithelial-to-mesenchymal transition (EMT) in experimental models [44]. Receptor-interacting protein kinase 3 (RIPK3) interacts with the inflammasome complex and amplifies profibrotic signaling, while pharmacologic RIPK3 inhibition reduced structural remodeling in vivo [45]. These findings position the RIPK3–NLRP3 axis as a promising target for combined anti-inflammatory and antifibrotic strategies.

Metabolic reprogramming further contributes to mesothelial transformation. Modulation of hypoxia-inducible factor 1 alpha (HIF-1α) via the mechanistic target of rapamycin and O-linked N-acetylglucosamine transferase (OGT) pathway mitigated MMT under high-glucose conditions [46]. Angiotensin receptor–neprilysin inhibition similarly limited EMT and M2 macrophage polarization through interference with TGF-β/Smad3 and signal transducer and activator of transcription 6 signaling [47]. These observations support a growing therapeutic paradigm targeting metabolic–immune crosstalk.

Adaptive immune subsets also participate in membrane remodeling. Peritoneal mucosal-associated invariant T cells drive mesothelial metabolic activation through MHC class I-related protein 1-dependent signaling and mechanistic target of rapamycin complex 1 activation, promoting fibrotic remodeling; pathway inhibition attenuated structural injury in experimental models [48]. Collectively, these data indicate that effective antifibrotic strategies will likely require multi-pathway modulation rather than single-target interventions.

### 7.2. Anti-Biofilm and Device-Directed Strategies

Given the role of the Tenckhoff catheter as a persistent microbial niche, targeting the biofilm device interface represents a complementary translational strategy. Biofilms protect pathogens from eradication and sustain low-grade immune activation that may perpetuate subclinical inflammation.

Surface engineering approaches aimed at reducing bacterial adhesion have shown promising experimental results. Nanostructured coatings incorporating anticoagulant and anti-adhesive components reduced bacterial attachment and biofilm formation on silicone substrates, highlighting the feasibility of device-level prevention strategies [49].

The bidirectional interaction between neutrophil extracellular traps and biofilms adds further complexity. While neutrophil extracellular traps contribute to antimicrobial defense, extracellular DNA may stabilize biofilm matrices and promote chronic infection persistence [50]. This dual role underscores the need for balanced therapeutic approaches that disrupt biofilm stability without impairing host defense. Combining catheter surface optimization with targeted biofilm-disruptive approaches may reduce the persistent inflammatory stimulus linking device colonization to progressive membrane remodeling.

### 7.3. Modulation of Mesothelial–Stromal Crosstalk

Preservation of membrane integrity likely depends on interrupting maladaptive intercellular communication within the peritoneal microenvironment. Extracellular vesicles have emerged as mediators of mesothelial–fibroblast signaling. Injured mesothelial cells release extracellular vesicles enriched in integrin-linked kinase (ILK), which activate fibroblasts through p38 MAPK signaling and promote fibrogenesis [51]. Inhibition of vesicle release attenuated fibroblast activation and reduced fibrosis in experimental models, while ILK-positive vesicles in dialysate correlated with membrane dysfunction. Targeting extracellular vesicle signaling may therefore represent a novel antifibrotic strategy with potential diagnostic and therapeutic implications.

### 7.4. Biomarker-Guided Molecular Stratification

Translation of mechanistic insights into clinical practice requires biomarkers capable of identifying patients at risk for progressive membrane dysfunction before irreversible structural change. PD effluent proteomics has revealed signatures associated with inflammatory activation and fibrosis [52]. Molecules such as alpha-Klotho (α-Klotho) correlate with baseline membrane status and fibrosis risk, suggesting that susceptibility to remodeling may precede dialysis initiation [53].

In pediatric populations, neutrophil extracellular trap components accumulate in dialysate and correlate with structural remodeling and transport characteristics, supporting their role as both mechanistic contributors and measurable indicators of membrane stress [15]. Future biomarker-driven approaches may enable early identification of patients at risk and support individualized therapeutic strategies. An integrated biomarker framework incorporating inflammatory mediators, extracellular vesicle cargo and metabolic signatures may enable more precise stratification and targeted antifibrotic or immunomodulatory interventions. However, translation into clinical practice will require validation in pediatric-specific cohorts, given the developmental modulation of immune and fibrotic responses.

## 8. Conclusions

Peritoneal membrane failure in pediatric PD does not arise from a single isolated insult but reflects the cumulative biological impact of repeated host–pathogen interactions within a chronically conditioned immune environment. Mesothelial cells, resident macrophages, recruited neutrophils, as well as adaptive immune subsets function within a milieu shaped by bioincompatible dialysate exposure, metabolic stress and persistent device-associated biofilms. Acute peritonitis episodes amplify pre-existing inflammatory circuits, while sustained signaling through innate immune receptors, inflammasomes, metabolic regulators and profibrotic transcriptional pathways progressively drives structural remodeling of the peritoneal membrane. In children, prolonged dialysis vintage during critical developmental periods may further intensify the long-term consequences of this inflammatory priming.

A unified conceptual framework that integrates pathogen-specific immune activation, biofilm biology, mesothelial–stromal communication and molecular drivers of fibrosis offers a more comprehensive explanation of technique failure than models focused solely on infection. Progress in this area will hinge on timely molecular stratification, focused modulation of inflammatory and fibrotic pathways, along with practical device-based approaches aimed at minimizing ongoing immune stimulation. Such an approach may move PD beyond infection control alone and support more durable, individualized membrane preservation in pediatric patients. Future research integrating longitudinal molecular biomarkers with mechanistic experimental models may enable earlier identification of vulnerable patients and facilitate the development of membrane-preserving therapeutic strategies.

## Figures and Tables

**Table 1:** Pathogen-specific immune activation patterns and remodeling profiles in pediatric peritoneal dialysis-associated peritonitis.

Pathogen Group	Primary PRRs	Key Downstream Pathways	Dominant Immune Pattern	Remodeling Consequence	Clinical Behavior
Gram-positive bacteria	TLR2	NF-κB, MAPK	Compartmentalized inflammatory activation	Recurrent low-grade remodeling (biofilm-driven)	High salvage potential
Gram-negative bacteria	TLR4	Myeloid differentiation primary response 88-dependent and TIR-domain-containing adaptor-inducing IFN-β-dependent signaling; NLRP3 inflammasome	High-intensity endotoxin amplification	Acute membrane stress, potentially reversible	Intermediate severity
*Mycobacteria*	TLR2, NOD-like receptors	Th1 cytokine axis (IFN-γ, TNF-α)	Persistent granulomatous activation	Progressive remodeling	High catheter removal
Fungi (*Candida* spp.)	Dectin-1, TLR2	NF-κB; Syk–CARD9 signaling	Biofilm-driven inflammatory persistence	Progressive membrane compromise	Very high catheter removal
Fungi (*Aspergillus* spp.)	Dectin-1	Syk–CARD9 signaling	Structural invasion beyond inflammatory containment	Necrosis + severe fibrosis	Highest mortality risk

**Table 2:** Molecular pathways linking inflammatory signaling to functional membrane failure.

Dominant Molecular Axis	Core Structural Effect	Functional Impact	Clinical Expression
PRR–NF-κB activation	Acute inflammatory infiltration	Increased permeability	Peritonitis episode
NLRP3–IL-1β signaling	Amplified leukocyte recruitment	High transport state	Fast transporter phenotype
NET persistence	Microvascular and stromal remodeling	Impaired free water transport	Early ultrafiltration decline
TGF-β1–MMT cascade	Collagen deposition, membrane thickening	Reduced ultrafiltration capacity	Progressive technique dysfunction
VEGF/HIF-driven angiogenesis	Increased capillary density	Accelerated solute absorption	Dialysis inadequacy
Chronic biofilm signaling	Sustained fibrotic activation	Irreversible architectural damage	Encapsulating peritoneal sclerosis

## Data Availability

No new data were created or analyzed in this study. Data sharing is not applicable to this article.

## Abbreviations

The following abbreviations are used in this manuscript:

PD	Peritoneal dialysis
EPS	Encapsulating peritoneal sclerosis
TLR	Toll-like receptor
NETs	Neutrophil extracellular traps
NETosis	Neutrophil extracellular trap formation
PRR	Pattern recognition receptor
PAMPs	Pathogen-associated molecular patterns
DAMPs	Damage-associated molecular patterns
NLRP3	NOD-, LRR- and pyrin domain-containing protein 3
NF-κB	Nuclear factor kappa B
IL	Interleukin
IL-1β	Interleukin-1 beta
TNF-α	Tumor necrosis factor alpha
TGF-β1	Transforming growth factor beta 1
VEGF	Vascular endothelial growth factor
HIF	Hypoxia-inducible factor
EMT	Epithelial–mesenchymal transition
MMT	Mesothelial-to-mesenchymal transition
eDNA	Extracellular DNA
MAIT	Mucosal-associated invariant T
BMSC	Bone marrow-derived mesenchymal stem cell
SDF-1	Stromal cell-derived factor 1
CXCR4	C-X-C motif chemokine receptor 4
ILK	Integrin-linked kinase
JNK	c-Jun N-terminal kinase
RIPK3	Receptor-interacting serine/threonine-protein kinase 3
Caspase-1	Cysteine-aspartic protease-1
